# The role of cancer‐associated fibroblasts in the tumour microenvironment of urinary system

**DOI:** 10.1002/ctm2.70299

**Published:** 2025-04-07

**Authors:** Ri Hong, Puguang Yu, Xiaoli Zhang, Peng Su, Hongyuan Liang, Dan Dong, Xuesong Wang, Kefeng Wang

**Affiliations:** ^1^ Department of Urology Shengjing Hospital of China Medical University Shenyang China; ^2^ Department of Critical Care Medicine Shengjing Hospital of China Medical University Shenyang China; ^3^ Medical Research Center Shengjing Hospital of China Medical University Shenyang China; ^4^ Department of Radiology Shengjing Hospital of China Medical University Shenyang China; ^5^ College of Basic Medical Science China Medical University Shenyang China; ^6^ Department of Urology People's Hospital of China Medical University Shenyang China; ^7^ Department of Urology People's Hospital of Liaoning Province Shenyang China

**Keywords:** cancer‐associated fibroblasts, exosomes, extracellular matrix, immune system, tumour microenvironment, urological tumours

## Abstract

Urological tumours are a type of neoplasms that significantly jeopardise human life and wellbeing. Cancer‐associated fibroblasts (CAFs), serving as the primary component of the stromal cellular milieu, form a diverse cellular cohort that exerts substantial influence on tumourigenesis and tumour progression. In this review, we summarised the literatures regarding the functions of CAFs in the urinary tumour microenvironment (TME). We primarily examined the multifaceted activities of CAFs in the TME of urological system tumours, including inhibiting tumour immunity, remodelling the extracellular matrix, promoting tumour growth, metastasis, drug resistance and their clinical applications. We also discussed potential future directions for leveraging artificial intelligence in CAFs research.

**Key points:**

- The interaction of CAFs with various cell secretory factors in the TME of urological tumors.
- The application of CAFs in diagnosis, treatment and prognosis of urological tumors.

## Graphical abstract

We summarised the role of cancer‐associated fibroblasts (CAFs) in urological tumours, including their origin, exosomes and extracellular matrix (ECM). CAFs are derived from various precursor cells and can communicate with other cells to form a hypoxic and immunosuppressive microenvironment. Additionally, CAFs can interact with tumour cells through various exosomes and exert a tumour‐promoting effect

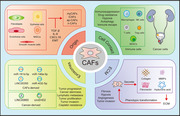

## 1 INTRODUCTION

Urinary system tumours primarily consist of renal cell carcinoma (RCC), bladder cancer (BCa) and prostate cancer (PCa), which are life‐threatening diseases that seriously impact human health. Based on the latest findings from the National Cancer Center, the estimated number of new cases of RCC, BCa and PCa in the United States in 2024 was 81 610, 83 190 and 299 010, respectively, exhibiting a prevalence that is consistently more pronounced in males compared to females.^1^ Although most patients with early‐stage malignant tumours of the urinary system have a good prognosis, individuals diagnosed with advanced or metastatic carcinomas is often associated with a markedly abbreviated survival duration, with the 5‐year survival rate for metastatic PCa remaining below 30%.^2^ Therefore, it is particularly important to find suitable targets in the tumour microenvironment (TME) to treat or delay tumour progression and recurrence.

**FIGURE 1:**
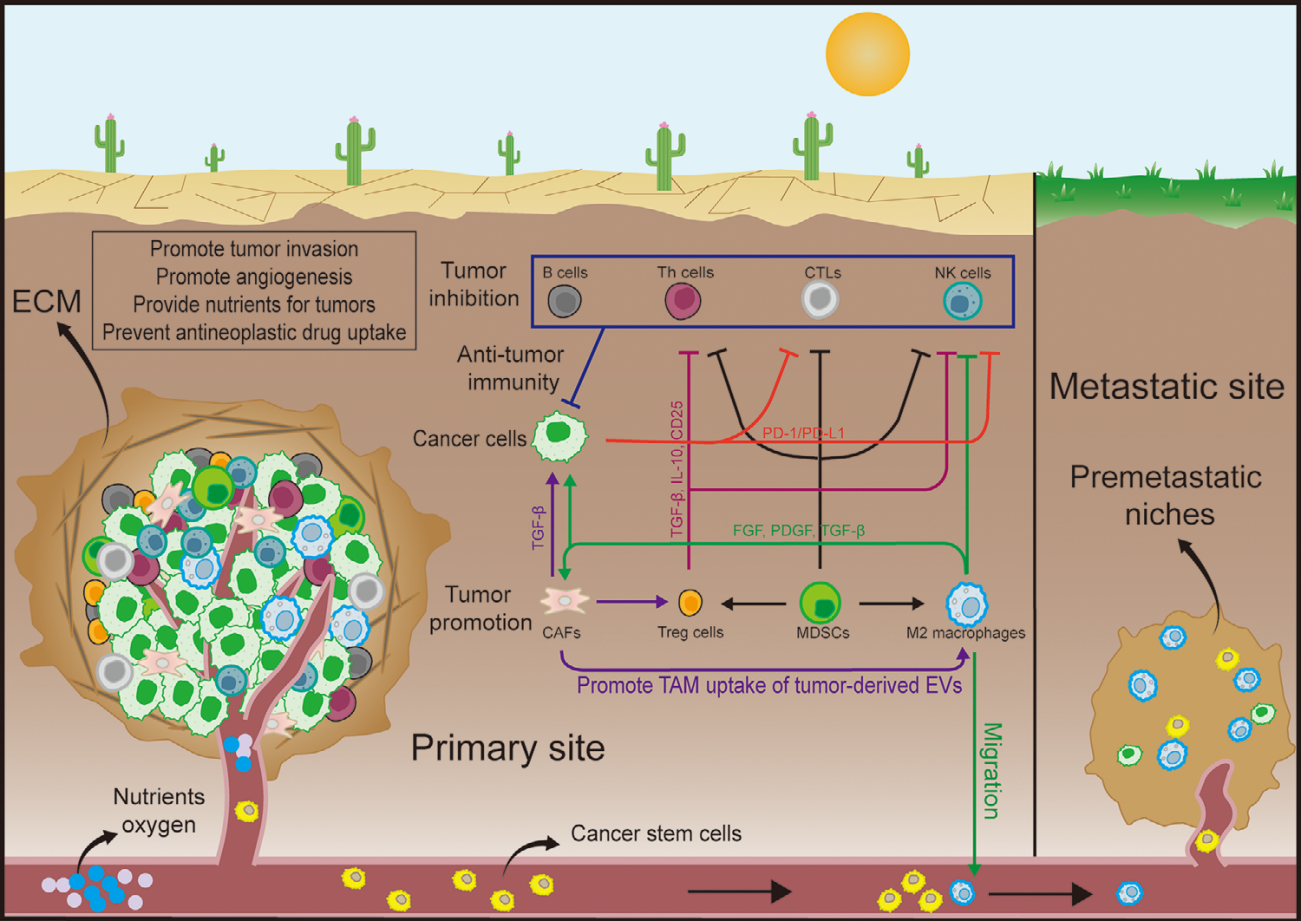
Cancer‐associated fibroblasts (CAFs) and tumour microenvironment (TME). The TME is an intricate ecosystem consisting of tumour cells, adjacent vasculature, stromal cells, immune and inflammatory cells, endothelial cells and their respective secretomes and extracellular matrix (ECM). This complex environment is the basic ecological niche that supports the existence of tumour cells. In this dynamic interaction, different cellular elements form a complex network of interactions that regulate the genesis and evolution of tumours.

The TME primarily consists of tumour cells and peripheral vessels, stromal cells, immune cells, inflammatory cells, endothelial cells and their secretory components, as well as the extracellular matrix (ECM). The individual components of the TME do not exist in isolation; instead, they are closely interconnected to form a complex network structure (Figure 1). Within the framework of the immunoediting hypothesis, the early TME facilitates the body's clearance of tumour cells. However, as tumours progress, the TME transforms into an immunosuppressive microenvironment, which enables tumours to evade the body's regulatory mechanisms and achieve immune escape.^3^, ^4^ The rapid proliferation of tumour cells, under the induction of genes such as hypoxia‐inducible factor‐1 (HIF‐1), contributes to the formation of a hypoxic microenvironment within tumours. This hypoxia can further activate NOTCH and transforming growth factor beta (TGF‐β) pathway, promoting angiogenesis and epithelial–mesenchymal transition (EMT).^5^, ^6^, ^7^ In response to external changes, cells within the microenvironment undergo metabolic reprogramming. For instance, tumour cells exhibit the Warburg effect, while glycolysis is enhanced in other cell types. The byproducts of this metabolic reprogramming further activate a series of signalling pathways. Recent findings indicate that lactate can serve as a substrate for protein lactylation, influencing tumour progression.^8^, ^9^ The TME, as the living environment for tumour cells, has consistently occupied a central position in research. The various processes of tumourigenesis and development are not solely driven by tumour cells but depend on the external TME. Cancer cells exhibit high heterogeneity and are prone to drug resistance, while the TME is relatively stable and more amenable to drug intervention, prompting more researchers to focus on the TME rather than exclusively concentrating on the tumour cells.^10^

Within the TME, various types of cells, including cytotoxic T lymphocytes (CTLs) and cancer‐associated fibroblasts (CAFs), have been found with roles that are not solely promoting or inhibiting tumour cell progression as traditionally thought. As research has delved deeper into these interactions, it has become clear that their effects on tumour cells are more nuanced and multifaceted.^11^ These cells exhibit functional heterogeneity based on their subtypes. For instance, CTLs are often associated with tumour killing and immune checkpoint therapy. However, they can also suppress anti‐tumour responses by lysing antigen‐presenting cells (APCs) or inducing macrophages to polarise towards an M2 phenotype, thereby promoting tumour progression.^11^, ^12^ CAFs are derived from multiple precursor cells that undergo transformation after experiencing the stimulus in the TME and are integral parts of the stromal cell population. Due to their varied origins and stimuli, they have different functions and heterogeneity. Their role in promoting or inhibiting the growth of different tumours is still debated; however, in the context of urinary tumours, they are known to promote tumour development. The conventional postoperative targeted therapy and inhibitors of urinary system tumours face challenges such as low response rates and drug resistance. As a major cell type in the matrix, CAFs participate in tumour‐related activities by participating in proliferation, invasion, EMT, metastasis, induction of angiogenesis and suppressing the immune function.^13^, ^14^, ^15^

In this manuscript, we reviewed the recent literatures published on the role of CAFs in urinary tumours TME to better understand the role of CAFs in urinary tumours and their potential as therapeutic targets.

## 2 ORIGIN AND SUBTYPES OF CAFS

### 2.1 Origin of CAFs

CAFs constitute a predominant cell population in the tumour stroma, and their origin remains a subject of debate. CAFs may originate from various precursor cells in the TME, including normal fibroblasts, pericytes, endothelial cells, epithelial cells, adipocytes and smooth muscle cells.^16^, ^17^ While fibroblasts are the primary source of CAFs, in a quiescent state, fibroblasts are activated by various stimuli in the TME and transformed into CAFs. Endothelial cells and epithelial cells can become CAFs through EMT.^18^ CAFs may also originate from recruited bone marrow mesenchymal stem cells (MSCs) by the TME under the influence of TGF‐β or CXCL16/CXCR6. This process enhances the expression of interleukin‐8 (IL‐8) and vascular endothelial growth factor (VEGF), thus promoting the growth of blood vessels within the TME.^19^ Upon entering the TME, MSCs begin to express molecules such as α‐smooth muscle actin (α‐SMA), fibroblast activation protein (FAP) and platelet‐derived growth factor receptor‐β (PDGFRβ), indicating that they have acquired a CAF‐like phenotype.^19^, ^20^, ^21^

The transdifferentiation of various precursor cells into CAFs, as well as the diverse stimuli received by normal fibroblasts at various locations within the TME, result in heterogeneity within the CAF population. Different subpopulations express unique markers and play distinct roles in the TME.^13^ CAFs also express both pro‐tumour and anti‐tumour signals in their cells, such as phosphatase and tensin homolog (PTEN) and c‐myc, which further demonstrates the diversity of CAFs.^22^ Additionally, the diversity of CAFs is regulated by multiple factors, including tumour progression, carcinogenic signalling from cancer cells and the ECM.^23^, ^24^

### 2.2 Subtypes of CAFs

Activated fibroblasts typically express vimentin, α‐SMA, FAP, S100A4 and PDGFRβ, however, these markers are not exclusive to CAFs but are also present in various other cellular populations. Markers specific to CAFs have not yet been identified. Based on their functions and marker expression, they are mainly categorised into myofibroblast‐like CAFs (myCAFs) and inflammatory CAFs (iCAFs). MyCAFs were originally considered to be the cells responsible for wound contraction and are commonly identified by α‐SMA and FAP. They serve as the primary source of the ECM, producing a significant amount of ECM proteins and exerting significant influence on the remodelling of the ECM. They are primarily modulated by TGF‐β signalling, which activates SMAD‐dependent and ‐independent pathways, participates in ECM remodelling, and promotes angiogenesis, inducing fibroblasts to transition to the myCAF phenotype.^25^ ICAFs express inflammatory factors including IL‐6, IL‐11, leukaemia inhibitory factor (LIF) and C‐X‐C motif chemokine ligand 12 (CXCL12), which promote tumour proliferation and are often correlated with adverse tumour outcomes.^26^ Under the influence of IL‐1, the downstream JAK/STAT pathway is activated, and CAFs are transformed into iCAF phenotypes with pro‐inflammatory activity that is dependent on NF‐κB signalling. TGF‐β counteracts this transformation by downregulating IL‐1R1, inducing fibroblasts to convert to the myCAF phenotype.^27^, ^28^

Another classification is based on the function of CAFs in tumours. Although CAFs generally show tumourigenic effects in urological system tumours, they are categorised into two main subtypes called cancer‐promoting CAFs (p‐CAFs) and cancer‐restraining CAFs (r‐CAFs), which are also evident in pancreatic and colorectal carcinoma.^29^, ^30^ Furthermore, the phosphoinositide 3‐kinase (PI3K)/protein kinase B (AKT) pathway, the wingless/integrated (WNT) pathway, mitogen‐activated protein kinase (MAPK) pathway and Notch homolog 2 (NOTCH2) pathway have all been thought to be implicated in the CAFs, influencing their functional diversity and activities.^25^, ^31^

Through single‐cell RNA sequencing (scRNA‐seq) and immunofluorescence methods, a novel subtype of CAFs was identified in BCa, named interferon‐regulated CAFs (ir‐CAFs). This subtype is induced by interferon beta (IFN‐β) and is characterised by upregulated expression of bone morphogenetic protein 5 (BMP5), neuregulin 1 (NRG1), stanniocalcin 1 (STC1), Wnt family member 5A (WNT5A) and the urea transporter solute carrier family 14 member 1 (SLC14A1). Activation of cyclic GMP‐AMP synthase (cGAS) and stimulator of interferon genes (STING) in tumour cells promotes IFN‐β production, leading to the development of ir‐CAFs. In ir‐CAFs, the WNT5A/β‐catenin pathway is upregulated, which enhances the tumour stemness of BCa and promotes tumour recurrence. In addition, ir‐CAFs are linked to unfavourable survival outcomes, resistance to chemotherapy and immunotherapy.^32^ Previously, scRNA‐seq has proved that antigen‐presenting cancer‐associated fibroblasts (ap‐CAFs) exhibit high expression levels of major histocompatibility complex class II (MHC‐II) and CD74, and phospholipase A2 group IIA (PLA2G2A) characterised CAFs in a highly metabolic state (me‐CAFs), primarily observed in the loose stroma of pancreatic carcinoma. Phenotypic changes can occur in different subtypes of CAFs, and various phenotypes may represent different functional states of the same CAF cell line.^33^, ^34^, ^35^

These new CAF subpopulations also play a role in immune regulation within tumours, either promoting or inhibiting anti‐tumour immunity, and could function as prospective biomarkers and targets for immunotherapy. The existence of different CAF subpopulations in various TMEs further illustrates the heterogeneity of CAFs.

## 3 ROLE OF CAFS IN THE TME

In the TME, CAFs do not operate independently but rather in collaboration with other cells and the ECM to create a network around the tumour. CAFs serve multiple roles, including the secretion of regulatory factors, promoting immunosuppressive conditions and angiogenesis, contracting smooth muscle, stimulating ECM reorganisation, collagen accumulation and promoting tumour proliferation and dissemination.^36^ Due to the heterogeneity of CAFs, subpopulations that express different molecular markers are capable of engaging with various cells and the ECM within the TME to execute their biological functions. Advancements in scRNA‐seq techniques and spatial transcriptomics have played a significant role in helping us identify the functional and spatial distribution differences among these subpopulations.

### 3.1 Interaction between CAFs and immune cells

Immune cells are key components of the TME. In the initial stages of tumourigenesis, under the influence of APCs, T lymphocytes, B lymphocytes, natural killer (NK) cells and macrophages within the TME exert their immune effector functions. Tumours can achieve immune evasion by downregulating surface antigens, exhibiting low expression of MHC molecules and secreting inhibitory factors. During this process, macrophages are continuously activated by the TME and gradually differentiate into the M2 phenotype, transforming into tumour‐associated macrophages (TAMs), which promote cell proliferation, angiogenesis and metastasis.^37^, ^38^

**FIGURE 2:**
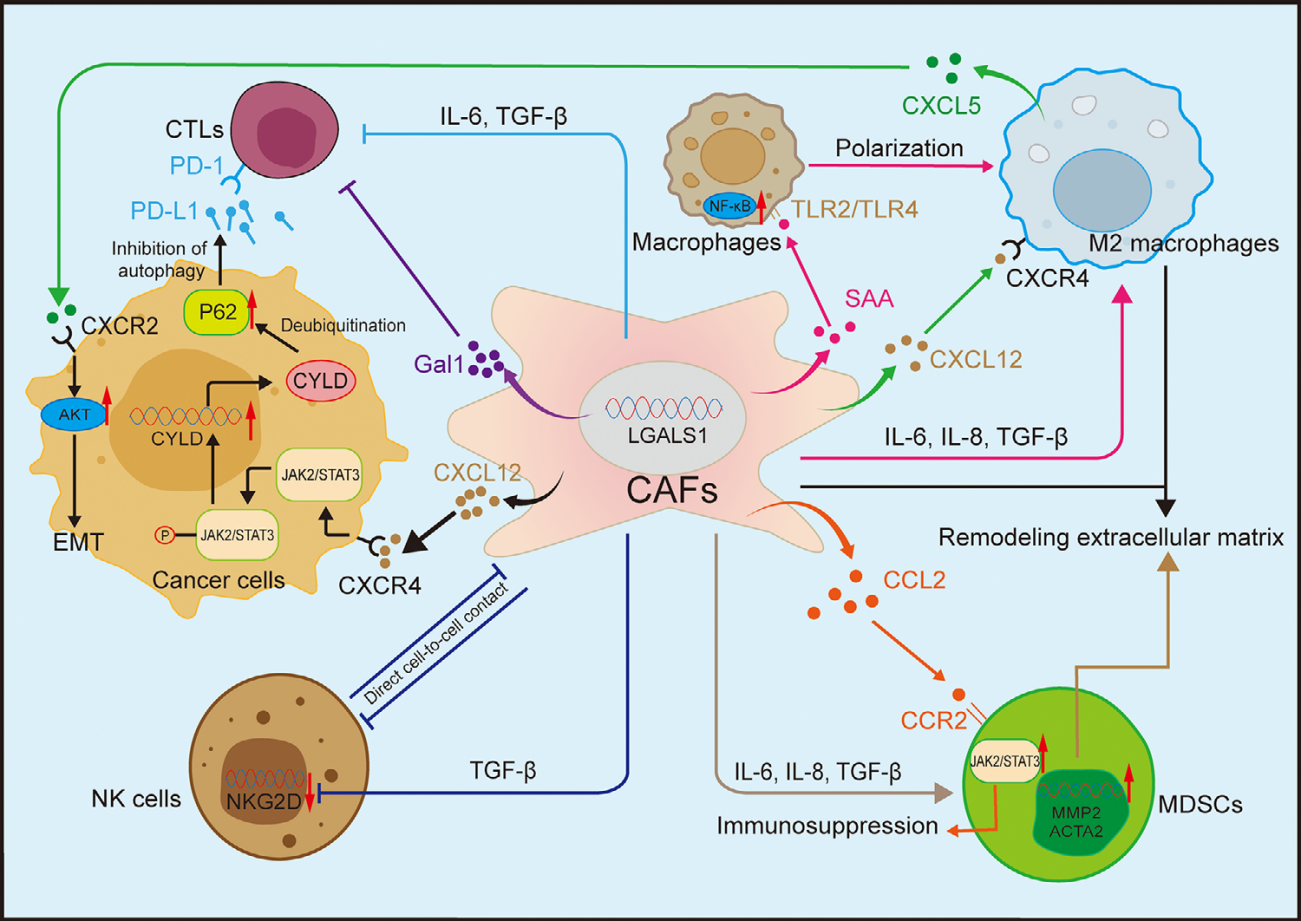
Interactions between immune cells and cancer‐associated fibroblasts (CAFs). CAFs can influence a variety of immune cells through the secretion of multiple factors. They can secrete mediators such as interleukin (IL)‐6, IL‐8 and transforming growth factor beta (TGF‐β) to promote or inhibit immune cell activity. In addition, they can secrete C‐X‐C motif chemokine ligand 12 (CXCL12), which acts on the CXCR4 receptor on tumour cells to upregulate cylindromatosis (CYLD) and promote the deubiquitination of P62. Deubiquitinated P62 inhibits autophagy of PD‐L1, leading to activation of the PD‐1/PD‐L1 pathway. Concurrently, CAFs can overexpress Gal1 and directly inhibit T cell activity. Serum amyloid A (SAA) secreted by CAFs can act on TLR2/TLR4 receptors and drive the polarisation of macrophages towards M2 phenotypes. CXCL12 can also act on the receptors of M2 macrophages, prompting them to secrete CXCL5, which binds to the receptors on tumour cells to enhance the AKT signalling pathway and lead to epithelial–mesenchymal transition (EMT). CAFs can also inhibit the function of NK cells through direct cell‐to‐cell contact. For myeloid‐derived suppressor cells (MDSCs), CAFs can secrete C–C motif chemokine ligand 2 (CCL2) to enhance the immunosuppressive function of MDSCs, upregulate MMP2 and ACTA2 in MDSCs, and cooperate with M2 macrophages to promote the remodelling of extracellular matrix (ECM).

Myeloid‐derived suppressor cells (MDSCs), predominantly exhibit immune‐inhibitory functions. They can activate regulatory T cells (Tregs) and M2‐TAM, promote EMT and angiogenesis, inhibit NK and CD8+ T cells, facilitating tumour immune escape.^39^ CAFs mainly act on immune cells and MDSCs by secreting cytokines and growth factors, inhibiting immune cell function or constructing inhibitory immune microenvironments to assist tumour immune escape (Figure 2). For example, CAFs secrete CXCL12 that can bind to its downstream receptors CXCR4 and CXCR7, activate downstream signals and participate in inflammatory diseases such as osteoarthritis and neuritis, as well as breast cancer, lung cancer and PCa.^40^, ^41^, ^42^ In BCa, CAFs‐derived CXCL12 interacts with CXCR4 and relies on the JAK2/STAT3 signalling pathway in cancer cells to upregulate the deubiquitinating enzyme cylindromatosis (CYLD), leading to the deubiquitination and accumulation of p62. This process inhibits autophagy of programmed death‐ligand 1 (PD‐L1), thereby suppressing the function of T cells and allowing tumours to evade immunity.^43^ In RCC, CXCL12 can also recruit TAM infiltration and affect tumour growth and invasion.^44^

Additionally, Peng et al.^45^ found that galectin‐1 (Gal1) was prominently upregulated in CAFs in RCC, particularly in recurrent cases. Gal1 is a cytokine with immunosuppressive properties that can promote T cell apoptosis and inhibit its cytotoxicity, and the expression of Gal1 is linked to reduced progression‐free survival (PFS). Immunohistochemistry also confirmed that T cell infiltration was significantly elevated in regions abundant in fibroblast activation protein‐positive (FAP+) CAFs, and the concentration of FAP+ CAFs in metastatic RCC (mRCC) was higher compared to primary RCC.^46^ This indirectly demonstrated that CAFs in RCC frequently interact with T cells, fostering the creation of a TME that suppresses immune responses, and that FAP+ CAFs are associated with mRCC.

In BCa, CAFs with elevated levels of nicotinamide adenine dinucleotide (NAD+) metabolising enzyme nicotinamide N‐methyltransferase (NNMT+ CAFs) can activate NF‐κB signalling in macrophages through serum amyloid A (SAA), transforming them into an M2‐like phenotype. They also polarise TAMs into tumour‐promoting phenotypes through the toll‐like receptor pathway involving TLR2/TLR4 and, together with TAMs, mediate ECM remodelling to form an immunosuppressive microenvironment that ultimately makes tumours resistant to the PD‐1/PD‐L1 immunotherapy pathways. NNMT+ CAFs may represent an excellent therapeutic target for BCa immunotherapy.^47^ Not only T cells and macrophages, but also in colorectal cancer, studies have found that CAFs can target and suppress the cytotoxicity of NK cells.^48^

In PCa, CAFs can simulate hypoxia signals in the TME, thereby activating HIF‐1 and its downstream pathways, influencing tumour metabolism and metastasis. Furthermore, activation of HIF‐1 induces CAFs to autocrinely secrete TGF‐β, promoting activation of myCAFs and secretion of CXCL13, a chemokine known to be involved in B cell recruitment.^49^, ^50^ In advanced PCa, B cells secrete lymphotoxin, which facilitates the aggravation of and tumour metastasis of castration‐resistant PCa (CRPC).^49^

Single‐cell sequencing has discovered a new CAF subpopulation in recurrent BCa: intercellular adhesion molecule‐positive infiltrating CAFs (ICAM1+ iCAF), which can secrete C–C motif chemokine ligand 2 (CCL2) binding to CCR2 on MDSCs, enrich STAT2 and NFKB2, recruit and activate MDSCs, and foster the TME characterised by immunosuppressive properties.^51^ Spatial transcriptomics also revealed ligand–receptor‐mediated crosstalk between CAFs and M2 macrophages in PCa, influencing the transformation to the M2 phenotype.^52^

In summary, in urinary system tumours, cellular interaction between CAFs and immune cells can create a pro‐tumour TME, thereby hindering the transport of therapeutic agents and the function of immune cells, and promoting immune escape.^53^

### 3.2 Interaction of CAFs with various cell secretory factors in the TME of urological tumours

#### 3.2.1 CAFs with cell secretory factors in the RCC TME

Originating from the tubular epithelial cells of the kidney, RCC is one of the most prevalent malignant kidney diseases in adults, with rising morbidity and mortality rates.^54^ Clear cell RCC (ccRCC) represents the predominant variant of RCC, constituting approximately 70%–80% of all documented cases. The pathogenesis of RCC involves deletion of the von Hippel–Lindau gene, substantial accumulation of lipids and glycogen, leading to induction of HIF, high expression of angiogenic factors including PDGF and VEGF, and upregulation of mechanistic target of rapamycin (mTOR) pathway.^55^

In the TME of RCC, CAFs can secrete pro‐tumourigenic factors including IL‐6, IL‐8, TNF‐α, TGF‐β and VEGF to enhance the proliferation, angiogenesis and metastasis of RCC.^56^, ^57^ IL‐6 and IL‐8, as cytokines, exhibit pro‐inflammatory and immunoregulatory effects, creating an inflammatory environment conducive to tumour development and establishing a foundation for tumour proliferation. The proliferation of tumours necessitates substantial amounts of oxygen and nutrients and depends on blood vessels to eliminate waste products. Under hypoxic conditions, VEGF is released by endothelial cells and binds to VEGFR2. With the aid of molecules such as delta‐like ligand 4 (DLL4) and angiopoietin‐2 (ANGPT2), VEGF stimulates angiogenesis, ensuring a sufficient oxygen and nutrient supply for tumours, and provides material conditions for the rapid development and metastasis of tumours.^58^, ^59^ CAFs communicate with tumours through TGF‐β signalling, promoting the recruitment of TAMs to metastatic sites, forming metastatic niches and providing an environmental basis for tumour metastasis.^60^ In ccRCC, the vasopressin 2 receptor (V2R) signalling pathway modulates various fibroblast regulators through the Yes‐associated protein (Yap) pathway, including IL‐8, CCL2, CCL5, colony stimulating factor 2, promoting the activation, proliferation and migration of fibroblasts.^61^

The spatial distribution of CAFs also influences their efficacy. In mRCC, CAFs located near tumour cells promote tumour proliferation, while tumour cells situated far from CAFs tend to undergo apoptosis.^62^ Spatial transcriptomics and multiple staining showed that myCAFs were predominantly distributed within the demarcation zone between tumour and adjacent non‐malignant parenchymal regions, occasionally invading normal tissue, and were spatially close to ccRCC, indicating close communication between the two cell types. They interact primarily through the TGF‐β pathway. Additionally, there are midkine (MDK)–low‐density lipoprotein receptor‐related protein 1 (LRP1), Jagged1 (JAG1)‐NOTCH, AXL receptor tyrosine kinase (AXL)–growth arrest‐specific 6 (GAS6) and IL‐6—IL‐6R pathways. The NOTCH pathway has previously been implicated in EMT, tumour stemness and CAF activation, while AXL‐GAS6 has been associated with immunotherapy resistance. These pathways promote tumour cell invasion and metastasis.^63^

RCC exhibits intrinsic resistance to radiotherapy and chemotherapy, with surgical excision serving as the standard therapeutic approach. For patients with advanced RCC (aRCC), molecular targeted therapy and immunotherapy are commonly used.^64^, ^65^ Angiogenesis inhibition is a pivotal therapeutic approach for treating aRCC, but recent literatures suggest that the quantity of CAFs is elevated significantly after VEGFR‐tyrosine kinase inhibitor (TKI). The proportion of CAFs within the tumour is often linked to shorter disease‐free survival and overall survival and diminishes the efficacy of VEGFR‐TKI.^56^ Therapeutic agents targeting immune checkpoint pathways have emerged as a cornerstone in the management of mRCC and have also been found to be associated with myCAFs resistance.^63^

HIF‐induced mTOR is one of the core mechanisms of RCC, so rapamycin (an mTOR inhibitor) is frequently used to treat RCC. Co‐culture experiments showed that CAFs continuously activated mTOR and conferred resistance to rapamycin in tumour cells.^66^ We investigated CAFs’ functions in promoting the spatial distribution of RCC and tumour chemoresistance. However, although CAFs induce resistance through multiple mechanisms, the specific molecular mechanisms remain unclear due to the lack of in vivo experimental validation. Currently, this knowledge cannot be translated into practical clinical application, and there are certain limitations. We believe that future drug development should start with the mechanisms of CAFs to reverse their tumourigenicity and drug‐resistant induced adverse effects.

#### 3.2.2 Interaction of CAFs with various cell secretory factors in the TME of BCa

BCa ranks as the 10th most common carcinoma globally, with 70% non‐muscle invasive BCa (NMIBC), 25% muscle invasive BCa (MIBC) and 5% metastatic BCa (mBCa).^67^ Currently, the primary treatment modalities are partial cystectomy, often supplemented by adjuvant chemotherapy based on cisplatin or transurethral resection of BCa, or radical cystectomy when bladder preservation is not feasible.^68^ However, MIBC is linked to a high metastasis rate, and its 5‐year survival rate remains less than favourable.^69^, ^70^ Even after treatment, BCa has a 50% recurrence rate.

In urinary system tumours, CAFs promote tumour metastasis primarily by secreting cytokines that activate various signalling pathways and induce EMT. EMT is the process by which carcinoma cells of epithelial origin undergo a phenotypic transition towards mesenchymal states through CAF‐mediated mechanisms. Neoplastic cells exhibit diminished structural polarisation and disruption of intercellular adhesive complexes, entering a state of low proliferation while gaining the ability to migrate and invade, thereby promoting tumour metastasis.^71^ In BCa, CAFs upregulate FAP through TGF‐β1, which subsequently affects the expression and secretion of versican (VCAN). The FAP/VCAN axis relies on the PI3K/AKT signalling pathway to promote EMT processes. TGF‐β1 can also augment the transcriptional levels of Zinc finger E‐box binding homeobox 2 (ZEB2) protein through TGF‐β1–long non‐coding RNA (lncRNA)‐ZEB2NAT transcription, further promoting the occurrence of EMT. In vitro experiments have confirmed that activated CAFs can induce EMT in cancer cells.^72^, ^73^, ^74^ CAF‐derived microfibril‐associated protein 5 (MFAP5) forms the MFAP5/NOTCH2 complex in a ligand‐independent manner, and indirectly activates the NOTCH2 signalling pathway through PI3K/AKT/ DLL4, promoting the malignancy, invasion and metastasis of BCa.^75^ Recently, Zheng et al.^76^ discovered the CAF subtype expressing PDGFRα and integrin alpha‐11 (ITGA11) on the membrane. ITGA11 activates the SRC/p‐VEGFR3/MAPK signalling pathway of lymphatic endothelial cells (LECs) by binding to selectin E (SELE) on LECs, leading to LEC reprogramming. They also observed that PDGFRα+ ITGA11+ CAFs in the tumours secreted chitinase‐3‐like protein 1 (CHI3L1), which led to a parallel arrangement of collagen in the ECM surrounding the carcinoma. Together, these two pathways promote early lymphovascular invasion and lymphatic metastasis of BCa.

In the TME, fibroblast growth factors (FGFs) bind to FGF receptor (FGFR) to activate the PI3K/AKT and rat sarcoma (RAS)/AKT pathways, which exert pivotal regulatory functions in tumourigenesis, angiogenesis, metastasis and TME regulation. Additionally, FGF/FGFR signalling is involved in regulating macrophage M2 polarisation and Treg cell survival. Inhibition of FGFR can reduce interferon‐gamma (IFN‐γ)‐mediated PD‐L1 expression, restore T cell function and promote anti‐tumour immunity.^77^, ^78^ A previous study indicated that activated FGFR signalling suppressed IFN‐γ‐mediated JAK/STAT signalling, thereby diminishing the expression of anti‐tumour immune‐related proteins including β2‐microglobulin, PD‐L1 and CXCL10.^79^ The previously mentioned intercellular adhesion molecule 1‐positive infiltrating cancer‐associated fibroblasts (ICAM1+ iCAFs) subtype has also been linked to promoting angiogenesis and EMT, thereby promoting BCa metastasis. It can secrete FGF2, act on the CD44 receptor in the recurrent cancer stem cell (rCSC‐M) subtype, promote the occurrence of recurrent BCa.^51^ Similar to BCa, it was reported in the literature as early as 2010 that PCa stem cells grow more aggressively when cultured with CAFs, indicating CAFs directly support their expansion.^80^ However, the literature on how CAFs interact with and affect cancer stem cells remains relatively limited. FGFR3 mutations can shorten patient survival, reduce T cell infiltration and cytotoxicity and create an immunosuppressive microenvironment that impairs clinical outcomes and treatment efficacy to immune checkpoint blockers (ICBs) in mBCa patients. Targeting mutated FGFR has shown hopeful directions in carcinoma, including BC, lung cancer and BCa, suggesting that the combination of FGFR‐TKIs and ICBs for metastatic urothelial carcinoma may be a promising treatment strategy.^81^, ^82^ CAFs also reduced the efficacy of PD‐L1 immunotherapy in BCa by secreting TGF‐β to repel T cells.^83^ In patients with basal‐type BCa, elevated FAP levels in CAFs were associated with enhanced muscle invasion and reduced patient survival, demonstrating its role in BCa deterioration and prognosis.^84^

In recent years, immune‐related CAFs (ir‐CAFs) demonstrate a significant correlation with tumour stemness, ICBs and the efficacy of chemotherapy. Although there is no direct evidence that ir‐CAFs are involved in the specific mechanism of these processes, it can be concluded that CAFs produce various classical growth factors and cytokines, including TGF‐β, FGF and others which mediate the invasion, EMT, metastasis, drug resistance and other processes associated with BCa. CAFs represent one of the pivotal constituent of the TME in BCa.

#### 3.2.3 Interaction of CAFs with various cell secretory factors in the TME of PCa

PCa relies heavily on androgen‐mediated androgen receptor (AR) cellular signalling, making hormonal regulation central to its biology and is the leading causes of morality among males globally.^67^, ^85^ Currently, there is no cure for CRPC, and there is a pressing requirement to discover new methods to prevent the occurrence of CRPC.

In PCa, CAFs play an important role in tumour progression through a variety of multiple mechanisms, depending on the AR status of the cancer cells. In classical PCa, CAFs can activate the AR signalling pathway to facilitate tumour progress. However, in AR‐negative cell lines, CAFs can still promote tumour growth and migration through paracrine TGF‐β1.^86^ In fact, TGF‐β1 stimulates the transformation of CAFs as early as the prostatic intraepithelial neoplasia stage, leading to increased type I collagen secretion and upregulation of FAP expression. This changes promote the development of reactive stroma, which progressively replaces the normal fibrous stroma as the tumour progresses.^87^ TGF‐β also influences the metastasis of PCa. Under the regulation of TGF‐β, CAFs and M2‐type macrophages upregulate CXCL12 and CXCR4, respectively, promoting the secretion of CXCL5 by macrophages and binding to the receptor CXCR2, upregulating the AKT signal pathway and facilitating the EMT of PCa.^88^

The TGF‐β type II receptor (TGFβR2) is also a subject of intense research. In 2012, it was discovered that loss of TGFβR2 on CAFs resulted in a lack of TGF‐β responsiveness and upregulation of IL‐8 and CXCL16, promoting castration resistance and bone metastasis in PCa.^89^, ^90^ Two years later, Banerjee et al.^91^ revealed that IL‐6 can lead to the deletion of TGFβR2 on CAFs, resulting in increased activity of DNA methyltransferase 1 (DNMT1) and histone H3 lysine‐9 trimethylation (H3K9me3), thereby damaging DNA repair genes and active oxygen metabolism genes, which promotes tumour progression. The researchers also examined the responsiveness of osteoblasts and osteoclasts to TGF‐β, and the disruption of it in osteoblasts promotes metastasis, potentially mediated by basic fibroblast growth factor (bFGF). Conversely, the loss of the TGF‐β signalling pathway in osteoclasts inhibits metastasis, although the specific mechanism behind this effect remains unknown.^92^

CAFs contribute to PCa proliferation and malignancy, which is closely associated with YAP1 activation. When YAP1 forms a complex with TEA domain transcription factor 1 (TEAD1), it promotes the transcription of downstream genes such as SRC, resulting in transforming the fibroblasts into CAFs, thereby increasing the aggressiveness of PCa.^93^ The activation of YAP1 not only facilitates this conversion but also changes CAFs from inhibiting tumour to promoting tumour. Specifically, activated YAP1 can transform lymphocyte‐associated CAFs (Lym‐CAFs), which typically exert an anti‐tumour effect, into ECM‐associated CAFs (ECM‐CAFs), which support tumour progression by modifying the TME. Importantly, studies have demonstrated that knocking out YAP1 can sensitise cancer cells which were not previously responsive to ICBs to anti‐PD‐1 therapy. This suggests that YAP1 inhibition could become a potential therapeutic approach for augmenting immunotherapy efficacy in PCa. Modulating the behaviour of CAFs through targeting YAP1 demonstrates significant potential for enhancing clinical outcomes and overcome resistance to existing therapies in patients.^94^

In PTEN‐deficient PCa, BMP2 and BMP7 can induce the expression of CXCL12 and inhibit the apoptosis of CAFs. Deacetylated Krüppel‐like factor 5 (KLF5) promotes the release of FGF9 by iCAFs through TNF‐α and promotes the secretion of CX3CR1 by tumour cells. FGF9 and CX3CR1 co‐activate FGFR1 and promote the progression of PCa.^95^, ^96^

**FIGURE 3:**
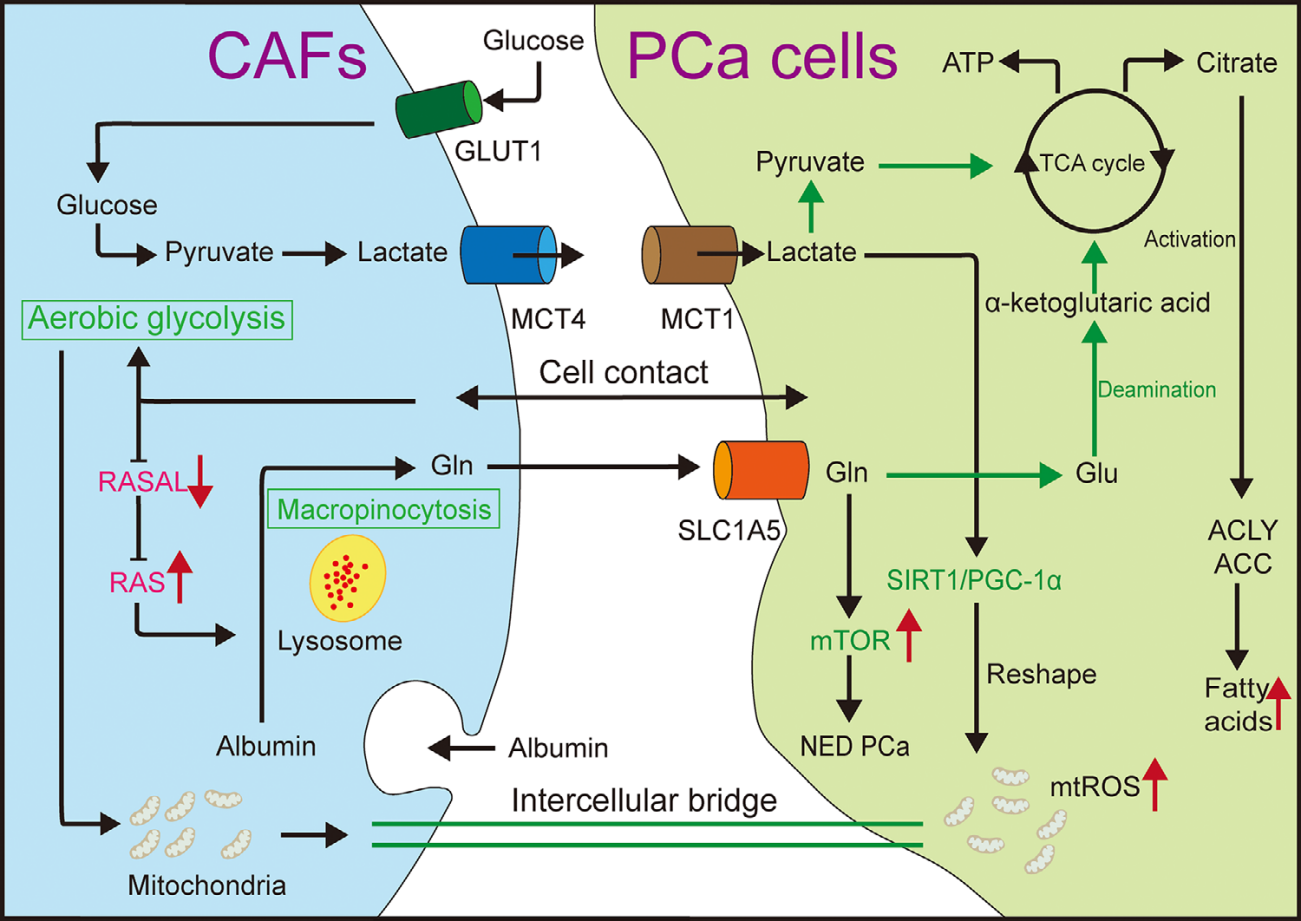
The symbiotic relationship between cancer‐associated fibroblasts (CAFs) and prostate cancer (PCa). PCa cells and CAFs coexist in a symbiotic relationship in the body. Once exposed, they undergo metabolic reprogramming. CAFs are converted to aerobic glycolysis to produce lactate, which is expelled from cells through monocarboxylate transporter 4 (MCT4), taken up by MCT1 and entered tumour cells. Additionally, CAFs in a high glycolytic state can provide excess mitochondria to tumour cells through intercellular bridge. Lactate entering tumour cells not only provides energy for tumour activity through tricarboxylic acid (TCA) cycle, but also upregulates key enzymes ATP citrate lyase (ACLY) and acetyl CoA carboxylase (ACC) in fatty acid synthesis through citrate pathway, promoting the production of lipid droplets and further exacerbating tumour progression. Moreover, lactate can activate the sirtuin 1 (SIRT1)/proliferator‐activated receptor gamma co‐activator 1‐alpha (PGC‐1α) pathway, leading to mitochondrial reshaping and the production of mitochondrial peroxides, which further increases the malignancy of tumours. Reprogramming of CAFs can also silence the RASAL gene, leading to an increase in macropinocytosis mediated by the rat sarcoma (RAS) signalling pathway and the production of more glutamine, which is taken up by SLC1A5. Glutamine that enters the tumour can participate in the TCA cycle by being converted to glutamate and undergoing deamination. It can also upregulate mechanistic target of rapamycin (mTOR), leading to the transformation of PCa into neuroendocrine subtypes and the development of resistance to androgen deprivation therapy (ADT).

In the microenvironment, CAFs and tumour cells can establish a symbiotic relationship (Figure 3). Upon contact with cancer cells, CAFs become activated, experience metabolic reprogramming and display the Warburg effect. They also utilise the upregulated monocarboxylate transporter 4 (MCT4) to extrude the lactate produced.^50^ Lactate released by CAFs is absorbed by tumour cells via MCT1, alters the internal nicotineamide adenine dinucleotide (NAD+)/NADH ratio, activates the sirtuin 1 (SIRT1)/peroxisome proliferator‐activated receptor gamma co‐activator 1‐alpha (PGC‐1α) axis, leads to mitochondrial reshaping and oxidative phosphorylation, promotes the accumulation of carcinogenic metabolites and the production of mitochondrial superoxide, and enhances tumour growth and metastasis. Additionally, lactate produced by CAFs induces lipid metabolism reprogramming in PCa, activating key enzymes, forming lipid droplets, providing a carbon source for histone acetylation and increasing invasiveness. CAFs also promote the upregulation of key enzymes 3‐hydroxy‐3‐methylglutaryl‐CoA synthase 2 (HMGCS2) and 3‐hydroxy‐3‐methylglutaryl‐CoA synaldo‐keto reductase family 1 member C3thase 2 (AKR1C3) in cholesterol and steroid synthesis through paracrine pro‐inflammatory molecules such as IL‐6 in tumour cells, increase cholesterol and steroid synthesis, provide additional energy and growth signals for cancer cells, and enhance resistance to AR‐targeted drugs. Simvastatin combined with AKR1C3 inhibitors can significantly suppress cancer cell proliferation. CAFs in contact with tumour cells will also be reprogrammed into an aerobic glycolytic state, and highly glycolytic CAFs can transfer excess mitochondria into reprogrammed tumour cells through gap junctions, further increasing the malignancy of PCa.^97^, ^98^, ^99^ In the TME, crosstalk between epithelial and mesenchymal components can lead to epigenetic silencing of RASAL3 in CAFs. Consequently, Ras promotes the synthesis of glutamine through macropinocytosis. CAFs enhance glucose consumption, promote glycolysis and switch to glutamine metabolism, thereby promoting metabolic reprogramming of cancer epithelial cells. Glutamine not only provides energy for tumour proliferation but also activates the mTOR pathway and facilitate tumour differentiation in neuroendocrine PCa, which is associated with androgen deprivation therapy (ADT) resistance. Moreover, ADT can exacerbate the silence of RASAL3.^22^

Currently, the primary treatment for PCa is ADT/AR signalling inhibitors (ARSIs), which aims to inhibit the transmission of AR signalling pathway.^85^ This treatment is frequently combined with chemotherapy, radiation therapy and/or immunotherapy to enhance its efficacy. However, following ADT/ARSI treatment, PCa inevitably progresses to CRPC. This mechanism may be related to CRPC development induced by secreted phosphoprotein 1 (SPP1+) myCAFs treatment.^100^, ^101^ ADT relies on TGF‐β signalling to transform iCAFs into SPP1+ CAFs, which promote the transformation of hormone‐sensitive PCa (HSPC) into CRPC through the SPP1–extracellular signal‐regulated kinase (ERK) paracrine pathway.^101^ For patients with CRPC, numerous growth factors and signalling molecules can activate AR without androgen, which may be a pivotal mechanism underlying the progression of CRPC. As previously noted, CAFs can directly activate the AR pathway through various signalling cascades. For example, IL‐6 produced by CAFs stimulates AR through the PI3K‐AKT, STAT3 and MAPK signalling pathways, thereby enhancing the malignancy of PCa cells. Further studies have revealed that IL‐6 can directly activate the PI3K‐AKT pathway in an AR‐independent manner, leading to increased VEGF secretion by PCa cells, which promotes angiogenesis in PCa. The precise mechanism underlying this has not been fully elucidated.^102^

Additionally, CAFs can produce and secrete glucosamine, which modifies ETS‐like protein 1 (ELK1) within tumour cells. The modified ELK1 promotes the transcription and increases the activity of 3β‐hydroxysteroid dehydrogenase type 1 (3βHSD1). As a key enzyme for extragonadal androgen synthesis, the enhancement of 3βHSD1 activity facilitates the efficient synthesis of androgens within the tumour, thereby promoting the progression of CRPC.^103^ Understanding how CAFs confer drug resistance on tumours is also an important research direction. In addition to affecting lipid metabolism, CAFs can secrete NRG1, which interacts with the HER3 receptor on tumour cells, thereby conferring sustained androgen resistance.^104^

Following ARSI treatment, CAFs can promote the transcription of androgen receptor variant 7 (AR‐V7) on PCa epithelium through IL‐6 and CD105‐mediated autocrine and paracrine BMP signalling pathways. AR‐V7, as a splice variant of AR, can transmit AR signalling pathway in the context of ligand deficiency and promote the progression of CRPC. Application of the CD105 antibody can mitigate resistance to ARSI, further validating this conclusion.^105^ Following docetaxel chemotherapy, it was found to promote mtDNA expression in PCa. PCa epithelial cells can secrete mtDNA, bind to dendritic cell‐specific intercellular adhesion molecule‐3‐grabbing non‐integrin (DEC205) on the membrane of CAFs, internalise mtDNA into CAFs, activate TLR9 and NF‐κB pathways, and stimulate the expression of complement C3. C3 was cleaved into C3a by the C3 convertase complex and released into the TME, increasing the resistance of PCa cells to docetaxel.^106^

Gemcitabine–cisplatin (GC) is commonly used as a combination therapy during docetaxel treatment for PCa. However, Eigentler et al.^107^ recently found that the expression of fibronectin 1 (FN1) and ITGA10 in CAFs increased after GC treatment of PCa. FN1 influences tumour invasion, adhesion and migration, while ITGA10 also affects CAF cell adhesion to the ECM and tumour growth. Alteration in the expression profile of CAFs also promotes ECM remodelling. In clinical practice, the use of GC and its implications remain a topic of debate.

We believe that CAFs are key elements in tumour drug resistance and hormone resistance. This phenomenon is also associated with metabolic reprogramming and immunosuppression within the TME. Targeting the inhibition and transformation of CAF function may significantly enhance conventional drug therapy. In addition, many researches have highlighted the indispensable function of CAFs in CRPC. CRPC is the predominant determinant of mortality among all patients with PCa, and currently no effective therapeutic options are available, resulting in a substantial disease burden. Targeting CAFs can inhibit or delay the onset of CRPC, improve therapeutic effect and prolong the overall survival of patients. For example, inhibiting the transformation of SPP1+ myCAFs and its paracrine mechanism can enhance the efficacy of ADT. In PTEN‐deficient PCa, the combination of CX3CR1 inhibitor and AKT inhibitor can significantly inhibit PCa growth.^95^, ^101^

#### 3.2.4 CAFs interact with various cell secretory factors in the TME through exosomes

**FIGURE 4:**
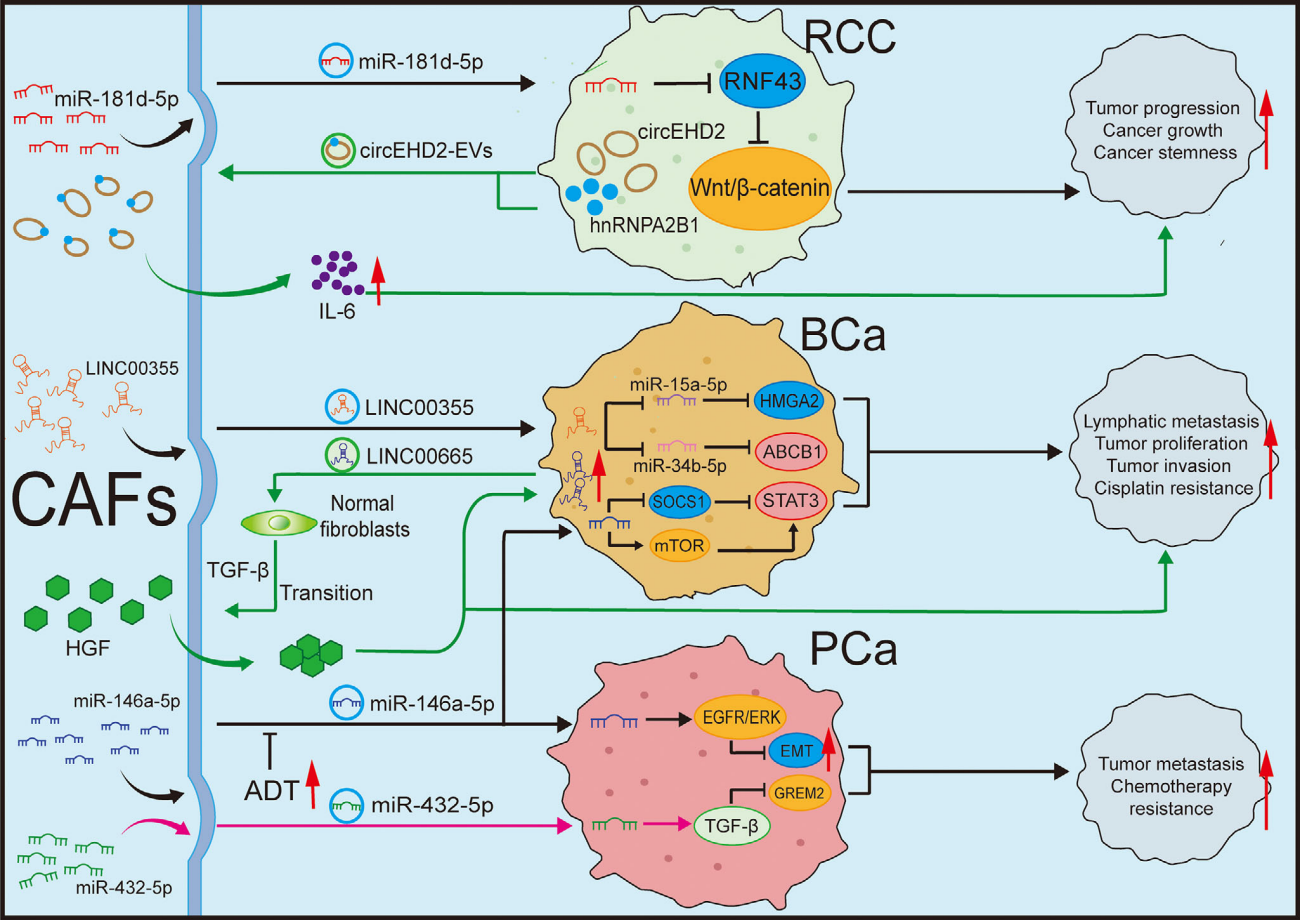
Exosomes between urinary system tumours and cancer‐associated fibroblasts (CAFs). Exosomes are important communication tools between CAFs and urinary system tumours. CAFs can encapsulate various signalling molecules in exosomes and deliver them to tumour cells to exert their effects. For instance, miR‐181d‐5p secreted by CAFs can directly suppress the expression of RNF43 in renal cell carcinoma (RCC) and activate the Wnt/β‐catenin signalling pathway, thus promoting the stemness and progression of tumours. CAFs can also secrete LINC00355, which competitively bind to miR‐15a‐5p in bladder cancer (BCa), leading to upregulation of high mobility group AT‐hook 2 (HMGA2). It can also bind to miR‐34b‐5p, increase the expression of its target gene ABCB1 and enhance the chemoresistance of BCa cells to cisplatin. miR‐146a‐5p released by CAFs targets suppressor of cytokine signalling 1 (SOCS1) and mechanistic target of rapamycin (mTOR) in BCa, leading to the activation of STAT3. miR‐146a‐5p has also been found to play a role in prostate cancer (PCa), where it can target the epidermal growth factor receptor (EGFR)/extracellular signal‐regulated kinase (ERK) pathway, affect epithelial–mesenchymal transition (EMT) and reduce the migration and invasion of PCa cells. CAFs secrete miR‐432‐5p, which targets GREM2 through the transforming growth factor beta (TGF‐β) pathway and promotes PCa resistance to taxanes. Similarly, tumour cells can also secrete exosomes that act on CAFs. RCC cells secrete circEHD2 vesicles that promote the activation of CAFs and interleukin (IL)‐6 secretion, thereby promoting the metastasis of RCC. LINC00665 secreted by BCa cells activates the classical TGF‐β signalling pathway, transforming fibroblasts into CAFs, stimulating CAFs to secrete hepatocyte growth factor (HGF), promoting lymphangiogenesis and lymphatic metastasis of BCa and ultimately leading to chemotherapy resistance.

Extracellular vesicles (EVs) can promote tumour proliferation, angiogenesis and lymphangiogenesis, and the establishment of a pre‐metastatic niche which promotes the colonisation of cancer stem cells in metastatic sites.^108^ Exosomes, as nanoscale vesicles typically spanning from 30 to 150 nm, enrich with a wide spectrum of biomolecules, encompassing nucleic acids, proteins and metabolites, that are used for intercellular communication and exert multifaceted influences in the TME.^109^, ^110^, ^111^ Here, we focus on the exosome transfer between tumours and CAFs (Figure 4). Exosomes deliver TGF‐β and promote the transformation of fibroblasts into myCAFs.^112^

In mRCC, the circular RNA EHD2 (circEHD2)/tyrosine 3‐monooxygenase/tryptophan 5‐monooxygenase activation protein (YWHAH)/YAP/SRY‐box transcription factor 9 (SOX9) signalling pathway accelerates the growth of RCC, and cancer cells can also secrete EVs containing circEHD2 to stimulates CAFs, causing them to activate and secrete IL‐6, thereby facilitating the metastasis of RCC.^113^ CAFs can also secrete exosomes, which regulate intercellular communication through paracrine mechanisms. Ding et al.^114^ pointed out that exosomes secreted by CAFs containing miR‐181d‐5p can directly reduce the level of ring finger protein 43 (RNF43) and activate the Wnt/β‐catenin signalling pathway, thus promoting the stemness and progression of cancer.

In BCa, exosomal long intergenic non‐coding RNA 00355 (LINC00355) derived from CAFs can competitively bind to miR‐15a‐5p, thereby upregulating high mobility group AT‐hook 2 (HMGA2) and promoting the proliferation and invasion of BCa cells.^115^ Subsequently, their team discovered that exosomal LINC00355 can confers cisplatin resistance in BCa through acting on miR‐34b‐5p to enhance the level of its target gene ATP‐binding cassette sub‐family B member 1 (ABCB1). As a competing endogenous RNA (ceRNA), exosomal LINC00355 plays an important role in chemotherapy resistance.^116^ Simultaneously, CAFs can also enhance the resistance of BCa to cisplatin and reduce cell apoptosis by modulating the insulin‐like growth factor 1 (IGF‐1)/oestrogen receptor beta (Erβ)/B‐cell lymphoma 2 (Bcl‐2) signalling pathway.^117^ Additionally, Li et al.^70^ revealed that LINC00665 recruits heterogeneous nuclear ribonucleoprotein L (hnRNPL) and induces histone 3 lysine 4 trimethylation (H3K4me3) modification on the Ras‐related protein Rab‐27B (RAB27B) promoter, leading to the upregulation of RAB27B at the transcriptional level, thereby increasing the secretion of EVs by BCa cells. In normal fibroblasts, EV‐mediated LINC00665 specifically promoted the phosphorylation of SMAD family member 2 (SMAD2) and 3 (SMAD3), activating the classical TGF‐β signalling pathway that converts fibroblasts into CAFs. CAF‐mediated C‐Myc can directly bind to the promoter of LINC00665, creating a positive regulatory pathway that continuously stimulates the transformation of CAFs. In this process, LINC00665 can also stimulate the production of hepatocyte growth factor from CAFs, promoting the genesis of lymphatic vessels and lymphatic spread of BCa.^70^ miR‐146a‐5p released by CAFs targets the 3′ untranslated regions (3′ UTR) of AT‐rich interaction domain 1A (ARID1A) and AMPKa2 mRNAs in BCa. This interplay acts on suppressor of cytokine signalling 1 (SOCS1) and mTOR, leading to the activation of STAT3, which ultimately enhances CSCs and impair the efficacy of chemotherapy.^118^

In PCa, CAFs secrete miR‐432‐5p, which inhibits chaC glutathione‐specific gamma‐lactamase 1 (CHAC1), thereby promoting the accumulation of glutathione, activating glutathione peroxidase 4 (GPX4) and preventing the increase of lipid‐derived reactive oxygen species. Consequently, ferroptosis and changes in mitochondrial membrane potential were inhibited, resulting in the resistance to docetaxel chemotherapy.^119^ Furthermore, exosomal miR‐423‐5p regulates gremlin 2 via the TGF‐β pathway, promoting resistance to chemotherapy in PCa.^120^ Zhang et al.^121^ discovered that exosomal miR‐146a‐5p from CAFs can also target the epidermal growth factor receptor (EGFR)/ERK pathway, influence EMT and prevent PCa from invading and migrating. Moreover, the decrease of miR‐146a‐5p by ADT in PCa can promote EMT transformation and PCa progression.

In summary, exosomes participate in many cancer‐related processes, such as EMT, tumour angiogenesis, ECM remodelling and chemotherapy resistance. Blocking the transport of CAF exosomes can inhibit tumour progression. For example, the application of antisense oligonucleotides can target the degradation of RNA within exosomes, potentially inhibiting their function.^122^ In 2020, Huang et al.^123^ developed a quantitative technique for detecting circulating exosomal PD‐L1 to facilitate cancer diagnosis and forecast the efficacy of immunotherapy. In mRCC, upregulation of circEHD2‐EVs in serum can be observed.^113^ Exosomes can be easily obtained from the body fluids of patients. Given the importance of exosomes in mediating communication between two types of cells, clinical diagnoses can be made based on the content of exosomes. Regarding treatment, exosomes serve as effective drug carriers for small molecular nucleic acids and chemotherapy agents due to their inherent properties of targeting tumour cells. This can minimise the side effects of drugs and enhance the therapeutic window. Previous studies have attempted to utilise exosomes to deliver chemotherapeutic agents targeting PC and breast cancer in vitro, and achieving promising efficacy.^124^, ^125^

### 3.3 Interaction of CAFs with ECM in the TME of urological tumours

CAFs not only communicate with the cellular components in the TME, but also significantly influence the ECM. The ECM is primarily composed of collagen, tenascin C, hyaluronic acid, fibronectin (Fn) and matrix metalloproteinases (MMPs). These macromolecules form a stable structure within the TME. Fibroblasts are instrumental in the process of wound healing. When injury occurs, inflammatory and immune cells migrate to the wound, secreting multiple cytokines which activate fibroblasts to form the ECM, which works to repair damaged tissue. However, as repair progresses to later stages, overactive fibroblasts continue to secrete ECM, primarily collagen, leading to an accumulation that can result in fibrosis at the wound site. In benign cases, this fibrosis is often closely associated with the progression of disease. For example, in benign prostatic hyperplasia (BPH), chronic inflammation drives the proliferation of prostate tissue and activates fibroblasts, leading to fibrosis. This increase in tissue stiffness worsens the symptoms of benign prostatic hyperplasia, especially lower urinary tract manifestations.^126^ Similarly, following acute kidney injury, renal tubular cells undergo oxidative stress and secrete pro‐inflammatory and pro‐fibrotic cytokines. These processes activate fibroblasts and promote fibrosis, eventually facilitating the transition from acute kidney injury to chronic kidney disease and even end‐stage renal disease.^127^, ^128^

Cancer can be compared to an obstinate wound, where the tumour promotes fibrosis, leading to stromal sclerosis and thickening of the ECM. MyCAFs represent the primary source of ECM in the TME.^129^ This process can be influenced by neuroendocrine factors. For example, under the influence of the sympathetic nervous system, tumours can produce inhibin βA, which in turn stimulates CAFs to produce collagens such as collagen type III alpha 1 (COL3A1), COL5A1, COL5A2 and COL11A1.^130^ Tumours often exist in a hypoxic environment, which can act on HIF‐1 and enhance the transcription of COL1A1. Collagen deposition is closely related to tumour development, histological grade and prognosis. For instance, the abundance of type I collagen in CAFs escalates in parallel with the advancement of BCa.^131^ Increased expression of type VI collagen is correlated with adverse prognosis and accelerated growth of ccRCC in vivo.^132^ COL11A1 exhibits elevated expression levels across multiple neoplastic entities, such as lung carcinoma, BC and BCa. Its overexpression is significantly correlated with unfavourable clinical outcomes and an increased propensity for metastatic dissemination.^133^, ^134^, ^135^ Picrosirius red staining revealed more pronounced levels of collagen deposition and fibrosis in the cortex and medulla of high‐grade ccRCC in contrast to low‐grade neoplasms.^136^

However, PCa presents a more complex scenario. In the early stage of PCa, the level of fibrosis within the stroma is relatively low. However, as tumours progresses from HSPC to CRPC, FAP was demonstrated to be positively correlated with disease progression. This marker is highly positive in advanced CRPC, with pre‐metastatic primary tumours showing elevated levels of CAFs, COL1A1, collagen type XII alpha 1 (COL12A1) and collagen type VI alpha 1 (COL6A1).^137^, ^138^ In PC‐3 PCa tumours, studies have shown lower fibre density in areas of severe hypoxia than in areas of moderate hypoxia, possibly due to increased urokinase‐type plasminogen activator receptor (uPAR)‐mediated COL1A1 degradation in areas of severe hypoxia, despite the fact that hypoxia promotes COL1A1 synthesis.^139^ Recent investigations have further underscored the role of YAP1 as a mechanotransducer capable of sensing changes in ECM stiffness, cytoskeletal tension and extracellular mechanical stress, thereby exacerbating the fibrotic process.^140^

The ECM serves as a critical determinant in regulating tumour invasion and angiogenesis. For example, in the TME of RCC, Fn can downregulate TGF‐β and inhibit tumour invasion and metastasis by interacting with SRC and SMAD phosphorylation.^141^ CAFs can reshape the ECM by producing ECM components, disrupting normal tissue structures and promoting tumour progression. The altered ECM can influence mechanical signal transduction, tissue hydraulic pressure, microenvironment acidification and mechanical rigidity of tumours in the TME, leading to the development of an immunosuppressive TME, reducing the uptake and efficacy of anti‐tumour drugs and supplying nutrients for tumour growth.^142^, ^143^, ^144^ CAFs can also secrete type I collagen, which interact with CD167a to enhance tumour cell invasion.^131^ Research has also indicated that the loss of COL1 can accelerate the progression of PCa. This finding highlights the heterogeneity of CAFs, suggesting that different subtypes of CAFs can exert both promoting and inhibitory effects on tumour development.^145^ Under the influence of non‐muscular myosin II, PDGFRα and α5β1 integrin, CAFs can also transform Fn from a reticular to a parallel fibre arrangement. Consequently, the tumour upregulates αv integrin and relies on the integrin for directed migration.^146^ They also produce MMPs, mainly MMP2 and MMP9, which promote EMT and thus facilitate tumour invasion and metastasis.^147^, ^148^, ^149^ Indeed, there have been initiatives to utilise artificial intelligence technology in combination with MMPs to diagnose prostate‐specific antigen (PSA)‐negative PCa.^150^

On the other hand, the ECM can also act in reverse on CAFs. For example, forkhead box F2 (Foxf2) in PCa stroma can induce the transformation of iCAFs to myCAFs, and can directly or indirectly downregulate CXCL5 transcription through NF‐κB or STAT3 pathways, reduce immunosuppressive myeloid cells, enhance the cytotoxicity of T cells and ultimately strengthen anti‐tumour immunity.^151^ The evolution of ECM is inextricably linked to tumour progression. Investigating ECM as a biomarker of cancer progression represents a critical research approach. Research in this area includes not only the search for new tumour markers in the ECM, but also the application of cutting‐edge technologies to stage and grade cancers accurately, quickly and non‐invasively. Research in this area includes not only the search for novel tumour markers in the ECM, but also the use of cutting‐edge technologies to stage and grade cancers accurately, quickly and non‐invasively, with the aim to tailor treatment regimens that improve patient outcomes. Additionally, the ECM plays a role in tumour immunology through mechanical interactions and various molecular mechanisms. Despite its importance, this area remains underexplored.

In addition, targeting the ECM presents an important anti‐cancer therapeutic strategy. Collagen is the principal component of the ECM and is involved in multiple intracellular signalling pathways, making collagen‐targeting drugs a promising avenue to explore. Notably, PCa can utilise CAFs to secrete lactic acid, which remodels collagen and enhances tumour aggressiveness.^152^ As a new substrate for protein modification, lactic acid has recently received significant attention. The specific mechanisms by which lactic acid is utilised in PCa, and whether similar mechanisms exist in the ECM of other tumours, require further investigation. This evidence suggests that the ECM can also serve as an important means for anti‐tumour therapy. Targeting inhibin βA against cachexia appears to be feasible in other carcinoma.^153^, ^154^ It may be a possible approach of anti‐tumour strategies by inhibiting or reversing the ECM remodelling by CAFs, or by reshaping the ECM.

## 4 CLINICAL APPLICATIONS OF CAFS IN UROLOGICAL TUMOURS

### 4.1 Application of CAFs in the diagnosis and treatment of urological tumours

It is evident that linking specific CAF subtypes to particular cancer subtypes can aid in patient stratification to develop personalised treatment, which suggests that CAFs research is clinical significance. This would not only improve therapeutic outcomes but also minimise unnecessary complications. Furthermore, characteristics of CAFs can guide the diagnosis, treatment and prognosis of patients. Recently, Boinapally et al.^155^ introduced the theranostic agent ^64^Cu‐FP‐L1, which targets tumours or lesions expressing FAP or prostate‐specific membrane antigen (PSMA) in the TME with high sensitivity, contributing to a more accurate diagnosis. Mona et al.^156^ also conducted clinical trials on 14 types of cancer patients using ^68^Ga‐FAPi‐46 PET and immunohistochemistry. This method successfully detected the expression of FAP in tumours and showed a close correlation with immunohistochemistry scoring (*r* = .783, *p* < .001). They determined that FAPi PET imaging can be utilised to detect FAP as an imaging diagnostic marker for patients.

We propose that future drug develop aimed at targeting CAFs should focus on activating CAFs, disease‐related proteins expressed by CAFs, drugs affecting CAF's communication with the TME or drugs specifically targeting CAF‐induced reprogramming or phenotypic changes.

Targeting CAF reprogramming to improve drug efficacy is an important research direction at present.^157^, ^158^ In 2017, Miao et al.^159^ exploited the off‐target effect of therapeutic nanoparticles to stimulate the secretion of tumour necrosis factor‐related apoptosis‐inducing ligand (sTRAIL) by CAFs. This therapeutic strategy demonstrated dual efficacy, effectively curbing the progression of tumours while simultaneously inducing the reversion of activated CAFs to a quiescent phenotype. In 2019, Lang et al.^160^ conjugated the FAP‐α‐specific antibody to cell‐penetrating peptide‐based nanoparticles to achieve targeted inhibition of CXCL12, inducing the stagnation of PCa. Some investigations have revealed that Am80 can augment the effectiveness of ICBs in PC and RCC. The expression of the rCAF marker Meflin and the production of Chemerin were stimulated to promote the conversion of pCAFs to rCAFs. Meflin+ rCAFs infiltration correlate with improved ICB outcomes for cases with ccRCC and urothelial carcinoma. Additionally, Chemerin promotes M1‐like polarisation of TAMs. The impact of Am80 on CAFs and TAMs enhances their response to PD‐L1.^161^ Therefore, the administration of Am80 to induce rCAFs prior to PD‐L1 treatment can effectively improve ICB efficacy. Subsequent treatment with recombinant Chemerin alone did not result in TAM polarisation, indicating that there may be other potential mechanisms involved in TAM polarisation induced by Chemerin that warrant further exploration. Recently, a clinical trial on pancreatic ductal adenocarcinoma demonstrated that the application of a TGF‐β receptor inhibitor, galunisertib, in combination with an autotaxin inhibitor, IOA‐289, could restore sensitivity to chemotherapy in mice. Similar observations were noted in patients.^162^ The FGF‐FGFR pathway, activated by CAFs, significantly impacts the development of various cancers, including BCa. Therefore, clinical studies targeting FGFR receptors have attracted considerable attention. After undergoing Phase II/III clinical trials, the FGFR inhibitor rogaratinib has shown therapeutic efficacy and safety comparable to chemotherapy for patients highly express FGFR1 and FGFR3 mRNA.^163^, ^164^ However, it was noted that the incidence of Grade 5 adverse events was more common in the rogaratinib group than in the chemotherapy group (16.3% and 6.1%). Although this difference was not statistically significant and was considered unrelated to the drug, further clinical trials should be conducted to verify the safety and efficacy of rogaratinib.

TLR4, a member of the toll‐like receptor family, serves as a crucial receptor for intracellular signalling pathways involved in inflammation and tumourigenesis. In PCa, cinnamaldehyde activates the TLR4‐dependent signalling pathway in CAFs, modifying their function and regulating downstream effectors, including JNK, TAK1 and c‐Jun. This promotes the transformation of CAFs to produce less immunosuppressant phenotypes, potentially enhancing immune responses in the TME.^165^ However, other studies have suggested that cinnamaldehyde may possess inhibitory properties targeting TLR4.^166^ Consequently, we believe that additional experiments are warranted to ascertain whether the anti‐tumour effects of cinnamaldehyde are concentration‐dependent and to elucidate the underlying mechanisms.

Autophagy enables the cell to degrade and recycle damaged organelles and misfolded proteins. This process is essential for maintaining cellular health and function. CAFs express high levels of ATG5 to sustain elevated autophagy, which promotes the development of PCa. Meanwhile, autophagy influences the secretion of cytokines and growth factors from CAFs, reshaping the TME and promoting tumour proliferation and metastasis. Research has demonstrated inhibiting ATG5 can slow the progression of PCa. These researches lay a theoretical groundwork for developing therapeutic strategies targeting ATG5, which may help improve treatment outcomes for PCa patients.^167^ We believe that drugs can be designed to target autophagy.

In summary, the examples discussed above suggest a potential approach for controlling the phenotypic transformation of CAFs or targeting CAF reprogramming to downregulate pro‐tumourigenic functions while activating anti‐tumour pathways. However, a current limitation is that many of these treatments remain in the laboratory stage and have not yet progressed to clinical trials. CAFs primarily activate the TGF‐β or IL‐6 pathways and secrete cytokines such as HGF and FGF. Targeting these pathways can inhibit CAFs activation and their tumour‐promoting functions. CAFs continuously secrete substances like collagen and Fn, remodelling the ECM and causing the TME to harden, which can prevent immune cells or drugs from being effective. Consequently, reversing this condition is a critical research direction. However, given that CAFs are heterogeneous and some subsets exhibit tumour‐suppressive functions, indiscriminate non‐specific inhibition of the TGF‐β pathway may lead to adverse therapeutic outcomes.

Additionally, recent discoveries indicate that anti‐arrhythmic drugs can reprogram CAFs through various mechanisms, such as regulating ion channels, reducing cell motility, inhibiting ECM remodelling and affecting communication between CAFs and PCa cells. This reprogramming creates an anti‐TME and suppresses the development of PCa.^168^ This opens up new possibilities for repurposing existing drugs. However, it is evident that the value of CAFs lies in their potential application in combination therapy. Currently, clinical trials involving CAFs in therapeutic strategies are primarily focused on combining them with ICB drugs to enhance treatment efficacy. This approach aims to leverage the synergistic effects between targeting CAFs and boosting the immune response against tumours, thereby overcoming the resistance mechanisms that tumours evade immune destruction. Inhibiting CAFs can suppress tumour proliferation to a certain extent, reverse tumour resistance to chemotherapy and immunotherapy, regulate the TME, and create an unfavourable environment for tumour development. Multi‐drug combination therapy based on CAFs blockade may yield unexpected effects in the future.

### 4.2 Application of CAFs in prognosis of urological tumours

CAFs have also been associated with the prognostic evaluation of urological malignancies. Mezheyeuski et al.^169^ stratified UC patients according with the expression profiles of five stromal biomarkers in CAFs and conducted a retrospective study. They discovered that FAP may serve as a vital prognostic indicator of unfavourable clinical response, and was dominant for CD90+ cases, CD8a cells with high expression were associated with a better prognosis.^169^ Furthermore, a cluster analysis of BCa patients indicated that those with high FAP+ CAFs had a lower 5‐year survival rate.^169^ However, a recent study aimed to compare FAP with PSMA as a diagnostic marker for PCa. They found that although FAP plays a partial substitution role in advanced PSMA‐negative CRPC, there is still a need for the discovery of new markers.^137^ We believe that enhancing the sensitivity of FAPi PET could improve the rate of FAP detection. Further studies across different tumour types are warranted to appraise the diagnostic potential of FAP in oncology.

Telomere length is also an independent prognostic factor for various tumours (including liver cancer, RCC and PCa) and their associated CAFs, and it is correlated with survival. Adenocarcinomas with longer telomeres exhibited more aggressive phenotypes.^170^, ^171^, ^172^ In senescent cells, the shortened telomere affects the EMT and exerts a significant influence on the progression of cancer.^173^ Telomere length reflects the trend of tumours and CAF immortalisation. The telomere length of CAFs is often related to that of tumours, suggesting that tumours may affect the progression of CAFs towards immortalisation by secreting certain factors or activating specific pathways. Moreover, we believe that telomeres are not only related to EMT and invasion during the tumour progression but may also participate in various tumour activities. We anticipate further researches in the future.

In addition to markers, CAFs could be further divided into multiple subtypes based on their transcriptional profiles, each serving a different biological function. Zhang and Liu^174^ identified genes with differential expression related to CAFs in response to radiotherapy, and derived two distinct gene signatures associated with CAFs. This method divided case reports into high or low risk cohorts, with the high‐risk cohorts demonstrating worse clinical outcomes, which successfully predicted their prognosis after radiotherapy.^174^ We look forward to the development of a more detailed classification of CAFs in urological system tumours to better understand their distinct roles, rather than simply categorising as myCAF or iCAF.

## 5 FUTURE PROSPECTS

**FIGURE 5:**
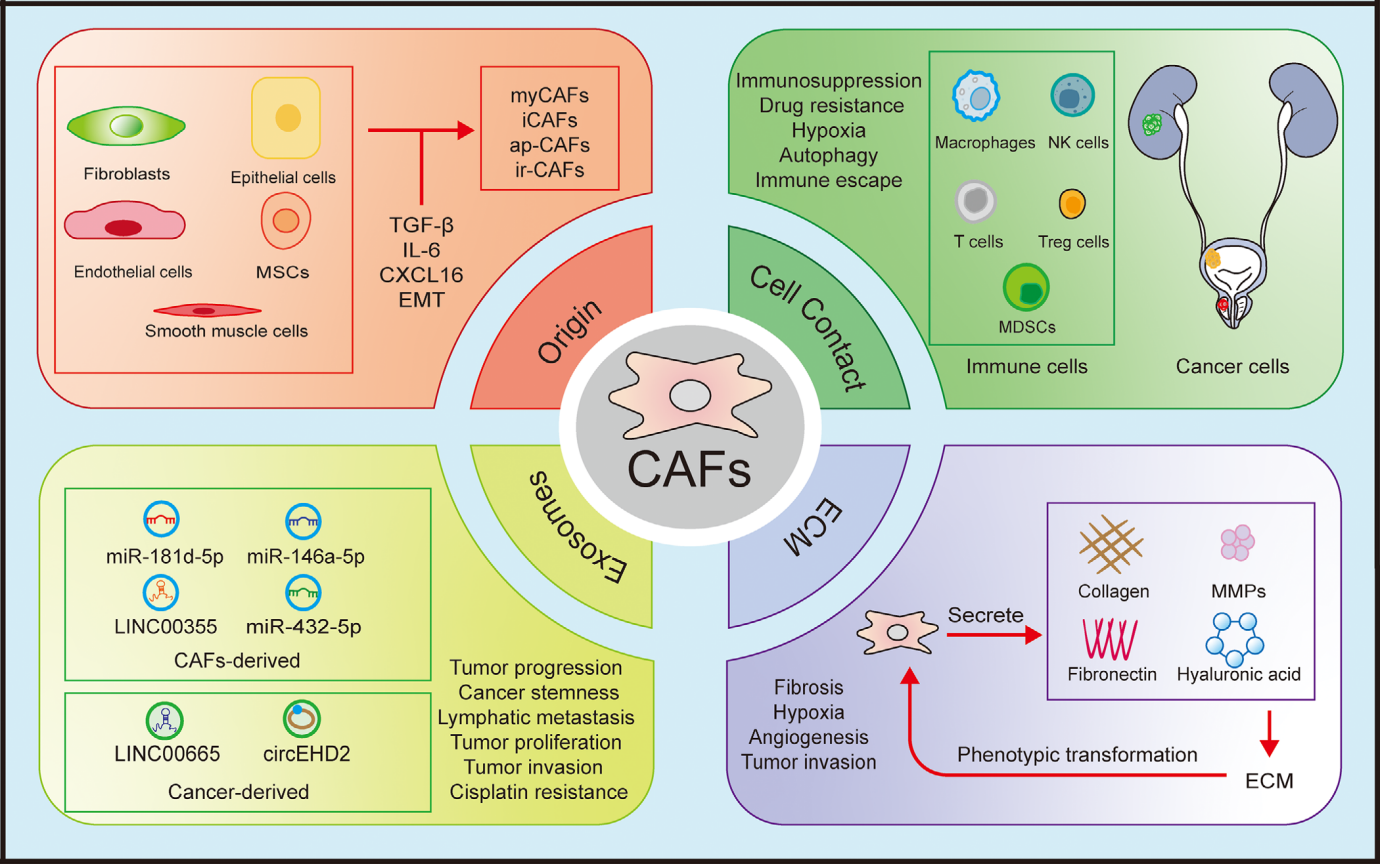
Graphical abstract of cancer‐associated fibroblasts (CAFs) in the tumour microenvironment (TME) of urinary system. The role of CAFs in urological tumours, including their origin, exosomes and extracellular matrix (ECM). CAFs are derived from various precursor cells and can communicate with other cells to form a hypoxic and immunosuppressive microenvironment. Additionally, CAFs can interact with tumour cells through various exosomes and exert a tumour‐promoting effect.

CAFs, as a vital cell type of the TME, participate in multiple aspects of the development of urological system tumours (Figure 5). As our understanding of the TME improves, CAFs will receive increasing attention, and clinical trials based on these cells are currently underway (Table 1).

**TABLE 1:** Clinical trials targeting cancer‐associated fibroblasts (CAFs).

NCT number	Study title	Conditions	Interventions	Study status	Phases
NCT03932565	Interventional Therapy Sequential With the Fourth‐generation CAR‐T Targeting Nectin4/FAP for Malignant Solid Tumours	Nectin4‐positive advanced malignant solid tumour	Biological: CAR‐T therapy for Nectin4‐positive malignant solid tumour	Unknown	Phase1
NCT04459273	Prospective Exploratory Study of FAPi PET/CT With Histopathology Validation in Patients With Various Cancers	Bladder carcinoma|Cervical carcinoma|Cholangiocarcinoma|Haematopoietic and lymphoid cell neoplasm|Hepatocellular carcinoma|Malignant adrenal gland neoplasm|Malignant brain neoplasm|Malignant pleural neoplasm|Malignant skin neoplasm|Malignant solid neoplasm|Malignant testicular neoplasm|Malignant thymus neoplasm|Neuroendocrine neoplasm|Thyroid gland carcinoma|Urothelial carcinoma|Cancer of unknown primary site	Procedure: Computed tomography|Drug: Gallium Ga 68 FAPi‐46|Procedure: Positron emission tomography|Radiation: 18F‐FDG	Recruiting	Phase1
NCT04504110	68Ga‐FAPI‐04 and 18F‐FDG PET/CT in Patients With Epithelial Ovarian Cancer: Compared With Histological Findings	Epithelial ovarian cancer	Diagnostic_Test: 68Ga‐FAPI‐04 PET/CT and 18F‐FDG PET/CT	Unknown	Phase2
NCT04621435	Imaging of Solid Tumours Using FAP‐2286	Solid tumours, adult|Metastatic cancer	Drug: Gallium‐68 labelled (68Ga‐) FAP‐2286|Procedure: Positron emission tomography (PET) imaging|Drug: Copper‐64 labelled (64Cu‐) FAP‐2286	Recruiting	Phase1
NCT04939610	A Study of 177Lu‐FAP‐2286 in Advanced Solid Tumours (LuMIERE)	Solid tumour	Drug: 68Ga‐FAP‐2286|DRUG: 177Lu‐FAP‐2286	Recruiting	Phase1|Phase2
NCT05064618	Investigator‐initiated Clinical Trial of MIKE‐1	Pancreatic cancer	Drug: Am80|DRUG: Gemcitabine|Drug: nab‐Paclitaxel	Recruiting	Phase1|Phase2
NCT05262855	Study of [68Ga]FAPI‐46 PET in Patients With Pancreatic Ductal Carcinoma	PDAC—pancreatic ductal adenocarcinoma|FAP	Drug: [68Ga]FAPI‐46	Recruiting	Phase2
NCT05323656	A Study of Setanaxib Co‐Administered With Pembrolizumab in Patients With Recurrent or Metastatic Squamous Cell Carcinoma of Head and Neck (SCCHN)	Squamous cell carcinoma of head and neck	Drug: Setanaxib|Biological: Pembrolizumab|Drug: Placebo	Active_Not_Recruiting	Phase2
NCT05518903	An Investigational Scan (68Ga‐FAPI‐46 PET/CT) for the Imaging of Cancer‐Associated Fibroblasts in Patients With Localised Pancreatic Ductal Adenocarcinoma	Localised pancreatic adenocarcinoma|Resectable pancreatic ductal adenocarcinoma|Stage 0 pancreatic cancer AJCC v8|Stage I pancreatic cancer AJCC v8|Stage IIA pancreatic cancer AJCC v8	Procedure: Computed tomography|Drug: Gallium Ga 68 FAPI‐46|Procedure: positron emission tomography	Recruiting	Phase2
NCT05547321	Efficacy and Safety Study of OMTX705, Monotherapy and Pembrolizumab‐combined, in Subjects With Advanced Solid Tumours.	Advanced solid tumour	Drug: OMTX705|Drug: Pembrolizumab	Recruiting	Phase1
NCT05641896	Study of [18F]FAPI‐74 PET in Patients With Gastrointestinal Cancers	Gastrointestinal cancers|Cholangiocarcinoma|Gastric cancer|Colorectal cancer|Pancreatic ductal adenocarcinoma|Hepatocellular carcinoma	Drug: [18F]FAPI‐74 PET/CT	Recruiting	Phase2
NCT05687747	Single Centre Prospective Evaluation of 68Gallium‐FAPI PET/MRI in Hepatocellular Carcinoma	Hepatocellular carcinoma	Drug: Radiolabelled tracer, 68Gallium‐FAPI‐46 PET/MRI	Not_Yet_ Recruiting	Phase1
NCT06107608	Molecular Imaging of FAP Expressing Cancer‐associated Fibroblasts in NSCLC Treated With Immune‐checkpoint Inhibitors	Non–small‐cell lung cancer	Procedure: FAPI PET/CT	Recruiting	Phase2
NCT06142318	Pirfenidone as a Radiosensitiser in the Treatment of Head and Neck Squamous Cell Carcinoma	Head and neck squamous cell carcinoma|Radiotherapy|Radiosensitiser|Pirfenidone	Drug: Pirfenidone|Drug: Placebo	Recruiting	Phase2

Given the heterogeneity of CAFs, they can exhibit tumour‐promoting or tumour‐suppressing characteristics in different tumours. However, advances in omics technology have facilitated our exploration of the internal structure of bioinformatics. We believe that in the future, utilising high‐throughput sequencing, such as scRNA‐seq and spatial transcriptomics will enable us to distinguish between CAFs that express different proteins and design drugs targeting specific CAF subtypes and their precise markers. A study has utilised single‐cell technology to classify CAFs within the PCa microenvironment into six subpopulations. It was found that these subpopulations have the function of recruiting inflammatory and immune cells by secreting chemokines such as CCL2 and CXCL12, providing new therapeutic targets for CAF‐targeted therapies.^175^ Single‐cell technology has revealed that NDUFA4L2+ myCAFs can form a physical barrier to isolate immune cells in breast cancer brain metastases. This finding highlights the role of these specific CAFs in creating a protective shield around tumour regions, thereby preventing immune cells from effectively attacking the tumour.^176^ As mentioned previously, CAFs can reduce the cytotoxic effects of CD8+ T cells, NK cells and macrophages in multiple studies. In pan‐cancer analyses including RCC and BCa, single‐cell sequencing has revealed that Biglycan secreted by CAFs is associated with poor prognosis and can predict the efficacy of ICB therapy.^177^ This further underscores the crucial role that advancements in sequencing technology play in exploring the heterogeneity of CAFs. Recently, a single‐cell analysis tool based on the Gene Expression Omnibus (GEO) database has been developed, which can be applied to eight types of tumours across 65 datasets. We believe that the development of this online tool lowers the barrier to entry for single‐cell technology, providing convenience for researchers analysing cells within the TME, including CAFs.^178^

Spatial transcriptomics can also assist us in investigating the internal structure of tumours. Research on CAFs often imposes the limitation that research models cannot fully replace the in vivo condition, which leads to inherent limitations in the conclusions. In two‐dimensional (2D) and three‐dimensional (3D) research models, the conclusions drawn may differ, sometimes even presenting opposite results.^5^ We believe that developing new co‐cultural models is also an important research direction. Recently, for example, a bio‐ink composed of 10% GelMA, 1% chondroitin sulfate and .1% HA was used to create co‐culture models of PCa and fibroblasts via 3D printing. Studies have found that hyaluronic acid has been demonstrated to modulate the secret of cytokines in carcinoma and indirectly induce the transformation of CAFs.^179^

CAFs can participate in clinical diagnosis, treatment and prognosis assessment; however, many experiments use FAP or α‐SMA as markers to identify CAFs. These biomarkers exhibit a lack of specificity for CAFs and are additionally detected in minute quantities within other cellular populations. We hope to conduce more relevant experiments in the future to identify molecules or proteins that are more representative of CAFs, which will provide us with broader insights into the functions of CAFs. We cannot overlook the significance of biomarkers in urological tumours. Multiple researches have discovered that the expression of FAP in ccRCC is closely linked to tumour aggressiveness, angiogenesis and metastasis. It is also related to tumour size, stage and grade in renal cancer. FAP serves as a powerful marker of tumour invasiveness and represents a promising therapeutic target.^180^, ^181^, ^182^ For instance, researchers are developing FAP‐activated prodrugs or FAP inhibitors, which have shown promising results in BCa and PCa cell lines as well as in mouse models. These studies provide a solid theoretical foundation and experimental basis for future applications in the treatment of urological tumours.^183^, ^184^

Additionally, current clinical trials based on CAFs are increasingly focusing on utilising PET CT for tumour stratification and on combining pathway inhibitors with ICB and chemotherapy drugs to improve treatment efficacy. For instance, a humanised monoclonal antibody named SRK‐181 has been designed to selectively inhibit TGF‐β1. This antibody not only improves the response of BCa to ICB therapy but also reduces the side effects that may arise from non‐specific inhibition of the TGF‐β pathway.^185^ We believe that more clinical trials should also focus on targeting the ECM remodelling by CAFs and the role of apCAFs in activating regulatory tregs. Given that CAFs are abundant in the stroma, their inherent physical functions within the TME also warrant investigation. Moreover, examining the differences between 2D and 3D model outcomes and attempting to explain these discrepancies could provide valuable insights.

Indeed, substantial knowledge deficits persist regarding the crosstalks between CAFs and CSCs. Because CSCs, as seeds, play a role in tumour metastasis, exploring their crosstalks with CAFs could yield novel perspectives on the underlying mechanisms of tumour spread. Research in this area can provide valuable perspectives on the complex mechanisms of cancer progression.

The progression of tumour immunotherapy is based on our comprehensive understanding of T, B, NK and other immune cells, fundamentally changing our perspective on tumours. Our knowledge of CAFs has not yet reached such levels of comprehensive understanding, as many mechanisms in the body are still unknown. We believe that investigating the fundamental mechanisms of CAFs is essential, as this knowledge will prove critical throughout the process of developing drugs to target CAFs.

## 6 CONCLUSIONS

In summary, we primarily discussed the functions of CAFs in the TME of urological cancers. CAFs interact with other TME cells through cytokines, chemokines and exosomes. Besides, they secrete ECM components like collagen and Fn, which influence tumour stiffness, activate signalling pathways and promote tumour progression. We also summarised the clinical applications of CAFs. They play a crucial role in clinical diagnostics, patient stratification, enhancing the efficacy of immunotherapy for patients. Given the dual role of CAFs in cancer promotion and suppression, reprogramming CAFs to shift from tumour‐promoting to tumour‐suppressing agents represents a significant potential therapeutic strategy. We hope that this article increases awareness of the roles of CAFs and provides new insights into cancer therapies targeting CAFs or the ECM.

## AUTHOR CONTRIBUTIONS

Kefeng Wang, Xuesong Wang, Dan Dong and Hongyuan Liang conceived the review; Peng Su and Ri Hong reviewed the information. Xiaoli Zhang and Ri Hong arranged the format of the figures. Ri Hong and Puguang Yu wrote the manuscript. Kefeng Wang, Xuesong Wang, Dan Dong and Hongyuan Liang critically reviewed the manuscript. All the authors read and approved the final manuscript.

## CONFLICT OF INTEREST STATEMENT

The authors declare no conflicts of interest.

## ETHICS STATEMENT

Not applicable.

## CONSENT FOR PUBLICATION

Not applicable.

## Data Availability

Not applicable.
